# A suggestion for quality assessment in systematic reviews of observational studies in nutritional epidemiology

**DOI:** 10.4178/epih.e2016014

**Published:** 2016-04-26

**Authors:** Jong-Myon Bae

**Affiliations:** Department of Preventive Medicine, Jeju National University School of Medicine, Jeju, Korea

**Keywords:** Meta-analysis, Quality evaluation, Research design, Quality control

## Abstract

**OBJECTIVES:**

It is important to control the quality level of the observational studies in conducting meta-analyses. The Newcastle-Ottawa Scale (NOS) is a representative tool used for this purpose. We investigated the relationship between high-quality (HQ) defined using NOS and the results of subgroup analysis according to study design.

**METHODS:**

We selected systematic review studies with meta-analysis which performed a quality evaluation on observational studies of diet and cancer by NOS. HQ determinations and the distribution of study designs were examined. Subgroup analyses according to quality level as defined by the NOS were also extracted. Equivalence was evaluated based on the summary effect size (sES) and 95% confidence intervals computed in the subgroup analysis.

**RESULTS:**

The meta-analysis results of the HQ and cohort groups were identical. The overall sES, which was obtained by combining the sES when equivalence was observed between the cohort and case-control groups, also showed equivalence.

**CONCLUSIONS:**

The results of this study suggest that it is more reasonable to control for quality level by performing subgroup analysis according to study design rather than by using HQ based on the NOS quality assessment tool.

## INTRODUCTION

Systematic reviews (SRs) with meta-analyses are a useful methodology to assess inconsistent epidemiological study findings [1]. However, controversy arose in the early 1990s regarding meta-analysis of the results of observational studies rather than randomized-controlled trials (RCT) [2-5]. Researchers have concluded that meta-analyses are not beneficial in cases where there are differences in the quality of the included studies [6,7]; in addition, the necessity for a scoring system to assess the quality levels of selected publications has also been emphasized [8,9].

The Newcastle-Ottawa Scale (NOS) is a representative tool developed for meta-analysis of observational studies [10]. The NOS is suitable for SR due to its easy application [11], and has been widely used due to recommendations from the Cochrane Collaboration [12]. Recently, however, the validity and reliability of the NOS has been questioned [13-15]. It was claimed to be additionally supplemented because the guidelines to be applied to each evaluation item were unclear [13,14]. Furthermore, Stang [13] suggested serious errors in the SRs that assessed the quality of observational studies using the NOS.

Since it is impossible to apply RCTs to nutritional epidemiology studies that investigate the relationships between daily food intake and the incidence of various cancers, only SRs for observational studies are available. Thus, Yang et al. [16] included ‘data analysis that used an energy-adjusted residual or nutrient-density model’ to the existing nine items of the NOS as an adjustment item to evaluate the quality of articles included in meta-analyses, which was then reflected to subgroup analyses. However, in their quality assessment [16], all three cohort studies were determined to be of high-quality (HQ), whereas only two of eight case-control studies were determined to be HQ. The reason for the difference in HQ determination between study designs appears to be attributable to the characteristics of the NOS, which was designed to give a higher score to cohort studies that are more scientifically persuasive [15].

Based on these observations, then, is it possible to replace the NOS tool with evaluating the quality level of observational studies in meta-analysis according to study design? It would be more reasonable and efficient if quality levels could be controlled according to study design rather than spending labor and time in applying the NOS. In other words, if there were consistency between HQ classification and study design, subgroup analysis by study design would be sufficient, instead of quality assessment using the NOS. Thus, the present study investigated the equivalence of meta-analysis results between HQ classification by NOS and cohort studies in same SR of observational studies on nutritional epidemiology.

## MATERIALS AND METHODS

### Subject article searches and selection criteria

The articles selected in the present study were those included in the analytical epidemiology SRs that investigated the relationships between daily food intake and the incidence of various cancers. In addition, they should use the NOS for quality evaluation and show the results of subgroup analysis by study design. The PubMed literature database (www.ncbi.nlm.nih.gov/pubmed) was used to search for articles using search terms corresponding to foods – diet, food, fruit, vegetable, or meat – and SR or meta-analysis for cancer incidence in the article title, abstract and keyword among lists published between January 2000 and October 2015.

After obtaining a list of articles, the following exclusion criteria were applied: (1) study hypotheses that did not assess the association between diet and cancer, (2) RCT rather than observational study, (3) SR without conducting meta-analysis, (4) SR without quality assessment, (5) SR having quality assessment by a tool other than the NOS, and (6) despite assessing quality using the NOS during the SR, subgroup analysis results were not included.

### Collection of related information and statistical analysis

Inclusion of articles in the analysis of quality assessment was based on statements in the methods section of each article, from which the type of tool used for evaluation and HQ decision criteria were identified. In order to determine the distribution of HQ subjects in each SR article according to study design, the articles were divided into cohort and case-control studies, and the statistical differences between the selected fractions (%) were examined using chi-squared tests.

In addition, the summary effect size (sES) and 95% confidence intervals (CI) estimated from the HQ group and cohort study group in subgroup analysis were extracted. The equivalence between the results of the HQ group and cohort study group was determined based on consistent direction of sES values to the null (=1) as well as consistent statistically significance based on the 95% CI.

## RESULTS

**Figure 1.**
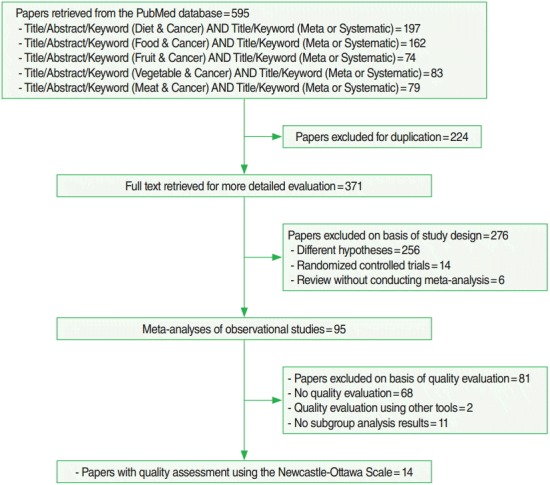
Flow chart of article selection.

Figure 1 shows the process of selecting final articles included in the analysis. After remove duplicate publications from the list obtained by searching formula, resulting in 371 articles identified for further review. Among them, (1) 256 articles were excluded because their study hypotheses did not assess the relationship between diet and cancer, (2) 14 articles were RCT, and (3) six articles performed SR without meta-analysis. Of the remaining 95 papers, 14 SR conducted quality assessments using the NOS as stated in their methods sections and also performed quality assessment in subgroup analysis [16-29]. Of those 14 articles, 4 [16-19] used modified NOS that included energy intake. Except for the study by Liu et al. [21], all others used seven points or more as the criterion for HQ. Table 1 presents results of HQ assessment as evaluated by the NOS according to cohort or case-control studies. Of the papers selected as subjects of meta-analysis in the 14 papers included in the current study, 81 (91%) of 89 cohort and 72 (34%) of 209 case-control studies were considered HQ, a statistically significant difference (p<0.01).

Table 2 summarizes the sES and 95% CI of the HQ and cohorts group by food item. The paper by Wang et al. [22] was excluded because it considered only cohort studies. Results of 19 datasets by food item from the 13 papers were summarized by food item; of these, 15 datasets showed the same magnitude and same statistical significance. The remaining four datasets lost statistical significance as CI became wide, although their magnitudes were consistent.

For the 15 datasets that showed equivalence between the HQ and cohort groups in Table 2, Table 3 was constructed to compare the results of subgroup analysis in the cohort and case-control study groups with overall sES by combining both results. Eight datasets showed equivalence in sES of the cohort and case-control study groups, and their overall sES also showed equivalence. In the seven datasets with non-equivalence, the results of case-control studies with higher article numbers greatly influenced the overall sES.

## DISCUSSION

In summary, the HQ and cohort groups had similar meta-analysis results because most cohort studies were classified as HQ based on quality assessment using the NOS. In other words, for SR of observational studies in nutritional epidemiology, quality assessment by NOS is decisively dependent on study methodology. Thus, subgroup analysis by study design may be more valid than the conducting quality assessment method based on the NOS, until a new quality assessment tool is developed in consideration of the characteristics of nutritional epidemiology.

As shown in Table 2, four datasets had no equivalence between the HQ and cohort groups. However, the width of CI changed while the magnitude of sES remained consistent, which resulted in their non-equivalence. Since the width of CI changes according to the number of subject articles included in the meta-analysis, the non-equivalence results was attributed to the difference in the number of subject articles, rather than differences between the HQ and cohort groups.

The current study also assessed whether non-equivalence could be controlled based on assessing results according to study design rather than the NOS. The results showed that when sES between the cohort and case-control groups showed equivalence, the overall sES for the combination of both group also showed equivalence. In addition, since the CI of the sES between the cohort and case-control groups in the paper by Hu et al. [29] was wide, there was no statistical significance; however, the CI of the overall sES of both groups combined was narrower, resulting in statistical significance. Thus, these findings suggest that it is reasonable to combine both groups when the sES calculated for each of the cohort and case-control groups shows equivalence. However, non-equivalence in the sES between these groups indicates that the overall sES should be interpreted carefully, as the value is dependent on the number of articles.

Colditz et al. [30] proposed that study design, quality of implementation, exposure, and covariates contribute to heterogeneity in SR. Since meta-analysis in nutritional epidemiology uses both cohort and case-control studies, there is heterogeneity by study design [31,32]. Therefore, while NOS evaluation within a group with the same study design would be meaningful, subgroup analysis only with the NOS while ignoring differences in study design may compromise the results.

In the present study, only 16.8% (=16/95) of papers (Figure 1) applied quality assessment results to subgroup analysis in their SR of nutritional epidemiology studies, even after including two papers that used their own quality assessment criteria instead of the NOS [33,34]. In addition, the first article to apply the NOS was published in 2006 [34], although the search included publication dates from January 2000. The primary reason for the lack of quality assessment in SR of nutritional epidemiology studies was the lack of valid assessment tools [33,34]. Although the NOS has been used since its development, the present study found that HQ decision in the NOS is fully dependent on study design. Thus, subgroup analysis should be performed separately for each study design until a quality assessment tool specific for nutritional epidemiology is developed.

The current study has several limitations. First, only the PubMed literature database was searched to investigate the level of quality assessment and a narrow set of terms related to diet items were applied to the search formula. In particular, items such as dietary fat, fiber, and vitamins indirectly assessed through diet measurement were excluded. Thus, the proportion as 16.8% (=16/95) (Figure 1) of papers that had subgroup analysis after quality assessment seems to be over-estimated. The results of the present study emphasize the importance of quality level control through subgroup analysis according to study design even without application of the NOS. Secondly, the equivalence of sES between the cohort and case-control study groups was comparatively analyzed based on study results that applied NOS. Thus, further investigations are necessary to determine if quality level can be controlled for in meta-analysis of nutritional epidemiology based on subgroup analysis results according to study design and application methods.

In conclusion, it is advisable to conduct subgroup analysis by study design for quality assessment of meta-analysis in nutritional epidemiology rather than applying the NOS assessment tool, and interpretation of overall sES should rely on the equivalence of sES by study design. These suggestions are consistent with the statement from Greenland [35] in 1994:

“Just as a diet and health study needs to examine the effects of each major dietary factor, quality scoring *should be replaced* by direct regression or stratification on objective quality-related study characteristics, such as *study design* (*cohort*, *case-control*, *etc*.), sources of data (direct interviews, mailed questionnaire, medical records, etc.), and sources of subjects (registry, hospital, etc.).”

## Figures and Tables

**Table 1.** Quality ratings of selected papers by study design

Author	YP	RN	Criterion of high quality	Cohort studies			Case-control studies		
				Selected [A]	High quality [B]	[B]/[A] (%)	Selected [C]	High quality [D]	[D]/[C] (%)
Yang et al.	2011	[16]	7+/10	3	3	100	8	2	25
Wu et al.	2013	[17]	7+/10	11	10	91	24	11	46
Wu et al.	2013	[18]	7+/10	6	6	100	16	5	31
Zhu et al.	2013	[19]	7+/10	12	10	83	30	13	43
Choi et al.	2013	[20]	7+/9	4	4	100	23	5	22
Liu et al.	2014	[21]	8+/9	2	2	100	31	4	13
Wang et al.	2014	[22]	7+/9	17	14	82	0	0	-
Yang et al.	2014	[23]	7+/9	10	9	90	9	2	22
Song et al.	2014	[24]	7+/9	4	4	100	14	8	57
Xin et al.	2015	[25]	7+/9	5	4	80	11	3	27
Wang et al.	2015	[26]	7+/9	6	6	100	13	7	54
Wu et al.	2015	[27]	7+/9	1	1	100	21	10	48
Li et al.	2015	[28]	7+/9	4	4	100	5	1	20
Hu et al.	2015	[29]	7+/9	4	4	100	4	1	25
Total				89	81	(91)	209	72	(34)

YP, year of publication; RN, reference number.

**Table 2.** Summary effect size (sES) and 95% confidence intervals (CI) of high quality groups on the basis of Newcastle-Ottawa Scale for quality assessment of cohort studies

Author [RN]	Food items	High quality		Cohort studies		Eq
		sES (95% CI)	NP	sES (95% CI)	NP	
Yang et al. [16]	Soy	0.70 (0.45, 0.99)	5	0.92 (0.85, 0.98)	3	Yes
Wu et al. [17]	Cruciferous vegetable	0.88 (0.80, 0.97)	21	0.93 (0.87, 1.00)	11	Yes
Wu et al. [18]	Cruciferous vegetable	0.84 (0.76, 0.93)	11	0.89 (0.77, 1.02)	6	No
Zhu et al. [19]	Red meat	1.30 (1.05, 1.61)	9	1.02 (0.90, 1.17)	4	No
	Processed meat	1.26 (1.10, 1.46)	17	1.18 (1.00, 1.38)	9	Yes
Choi et al. [20]	Red meat	1.60 (1.20, 2.13)	8	1.26 (1.00, 1.59)	4	Yes
	Processed meat	1.20 (0.88, 1.62)	6	1.25 (0.83, 1.86)	3	Yes
Liu et al. [21]	Vegetable	0.97 (0.78, 1.21)	3	0.91 (0.68, 1.21)	2	Yes
	Fruit	0.96 (0.69, 1.33)	2	0.81 (0.58, 1.12)	1	Yes
	Soy	1.12 (0.68, 1.84)	3	1.46 (1.07, 1.98)	2	No
Yang et al. [23]	Vegetable	0.68 (0.59, 0.78)	9	0.66 (0.51,0.86)	9	Yes
	Fruit	1.03 (0.87, 1.20)	7	1.04 (0.91, 1.20)	6	Yes
Song et al. [24]	Red meat	1.27 (1.09, 1.48)	17	1.00 (0.83, 1.20)	8	No
Xin et al. [25]	Vegetable oil	0.99 (0.87, 1.13)	7	0.94 (0.84, 1.06)	5	Yes
Wang et al. [26]	Cruciferous vegetable	0.61 (0.44, 0.86)	6	0.76 (0.62, 0.93)	6	Yes
Wu et al. [27]	Vegetable	0.78 (0.54, 1.14)	7	1.00 (0.52, 1.92)	1	Yes
	Soy	0.87 (0.60, 1.26)	5	1.09 (0.60, 1.98)	1	Yes
Li et al. [28]	Cruciferous vegetable	0.78 (0.55, 1.01)	5	0.87 (0.67, 1.05)	4	Yes
Hu et al. [29]	Cruciferous vegetable	0.89 (0.77, 1.02)	5	0.92 (0.80, 1.07)	4	Yes

RN, reference number; Eq, equivalent direction and statistical significance of sES between high quality group and cohort studies (yes or no); NP, number of papers.

**Table 3.** Summary effect size (sES) and 95% confidence intervals (CI) on the basis of overall and case-control studies about papers showing equivalence of direction and statistical significance between cohort and high quality group in Table 2

Author [RN]	Food items	Cohort studies		Case-control studies		Eq	Overall		
		sES (95% CI)	NP	sES (95% CI)	NP		sES (95% CI)	NP
Yang et al. [16]	Soy	0.92 (0.85, 0.98)	3	0.72 (0.56, 0.92)	8	Yes	0.77 (0.65, 0.92)	11
Wu et al. [17]	Cruciferous vegetable	0.93 (0.87, 1.00)	11	0.74 (0.65, 0.84)	23	Yes	0.82 (0.75, 0.90)	35
Zhu et al. [19]	Processed meat	1.18 (1.00, 1.38)	9	1.64 (1.47, 1.83)	17	Yes	1.45 (1.26, 1.65)	26
Choi et al. [20]	Red meat	1.26 (1.00, 1.59)	4	1.44 (1.16, 1.80)	18	Yes	1.38 (1.17, 1.64)	22
	Processed meat	1.25 (0.83, 1.86)	3	1.36 (1.07, 1.74)	15	No	1.32 (1.08, 1.62)	18
Liu et al. [21]	Vegetable	0.91 (0.68, 1.21)	2	0.67 (0.42, 1.06)	7	Yes	0.72 (0.51,1.02)	9
	Fruit	0.81 (0.58, 1.12)	1	0.63 (0.42, 0.94)	6	No	0.66 (0.47, 0.91)	7
Yang et al. [23]	Vegetable	0.66 (0.51,0.86)	9	0.76 (0.48, 1.20)	8	No	0.70 (0.56, 0.87)	17
	Fruit	1.04 (0.91, 1.23)	6	0.78 (0.61,0.98)	6	No	0.93 (0.80, 1.08)	12
Xin et al. [25]	Vegetable oil	0.94 (0.84, 1.06)	5	0.86 (0.72, 1.02)	11	Yes	0.87 (0.77, 0.99)	16
Wang et al. [26]	Citrus fruit	0.76 (0.62, 0.93)	6	0.54 (0.41,0.72)	13	Yes	0.63 (0.52, 0.75)	19
Wu et al. [27]	Vegetable	1.00 (0.52, 1.92)	1	0.76 (0.60, 0.96)	12	No	0.77 (0.62, 0.96)	13
	Soy	1.09 (0.60, 1.98)	1	0.66 (0.48, 0.92)	14	No	0.68 (0.50, 0.93)	15
Li et al. [28]	Cruciferous vegetable	0.87 (0.67, 1.06)	4	0.72 (0.55, 0.89)	5	No	0.78 (0.64, 0.91)	9
Hu et al. [29]	Cruciferous vegetable	0.92 (0.80, 1.07)	4	0.87 (0.73, 1.03)	4	Yes	0.89 (0.81,0.99)	8

RN, reference number; Eq, equivalent direction and statistical significance as sES between cohort and case-control studies (yes or no); NP, number of papers.
