# Supplementary material for: A suggestion for quality assessment in systematic reviews of observational studies in nutritional epidemiology
Source: Epidemiol Health. 2016 Apr 26;38:e2016014. doi: 10.4178/epih.e2016014 (PMC4877518; doi:10.4178/epih.e2016014)
Supplement: Supplementary file 1 [file epih-38-e2016014-supplementary.pdf]

<**Methods**>

A Suggestion for Quality Assessment in Systematic Review of Observational Studies for Nutritional Epidemiology

식이역학 관찰연구의 체계적 고찰에서 질적 수준 평가에 관한 제언

국문요약

관찰연구에 대한 메타분석을 수행할 때, 선정된 논문들의 질적 수준을 통제하기 위해 개발된 Newcastle-Ottawa Scale (NOS)는 평가 항목의 속성상 환자-대조군연구보다는 코호트 연구에서 High quality (HQ) 판정이 될 가능성이 높다. 이에, 식이와 암의 관련성을 연구한 관찰연구에 대한 메타분석에 있어 대부분의 코호트 연구를 HQ로 판정하였기에, HQ 군과 Cohort 군의 메타분석 결과는 동일하였다. 코호트군과 환자-대조군 간의 summary effect size (sES)가 동질성을 보일 경우 이를 합친 Overall sES 또한 동질성을 보였다. 이에, 식이역학에 특화된 질적평가도구가 개발되기 까지는 NOS를 적용하기 보다는 연구설계별로 하부군 분석을 하는 것을 권한다.

Introduction

메타분석을 적용한 체계적 고찰 (systematic reviews, SR)은 역학연구 결과들이 일관성이 없을 경우 유용한 연구방법론이다 [1]. 그러나 무작위임상시험 (randomized-controlled trials, RCT)이 아닌 관찰연구 (observational studies) 결과들에 메타분석을 시행하는 것에 있어 1990년 초에 많은 논란이 있었다 [2-5]. 그 중 연구들 간의 질적 수준의 차이가 있을 경우 메타분석은 유해하다는 주장 [6,7]과 함께, 선정된 논문들의 질적 수준을 평가하고 이를 반영하는 scoring system의 필요성이 강조되었다 [8,9].

The Newcastle-Ottawa Scale (NOS)는 관찰연구의 메타분석을 위해 개발된 대표적인 도구이다 [10]. NOS는 적용하기 쉬워서 SR에 사용하기 적합하며 [11], 코크란 연합 (the Cochrane Collaboration)에서 권장하였기 때문에 널리 사용되어 왔다 [12]. 그런데, 최근 NOS 도구에 대한 타당성과 신뢰성에 의문이 제기되었다 [13-15]. 특히 각 평가 항목 당 적용할 지침이 불명확해서 [13,14] 추가적인 보완을 해야만 한다는 것이다 [14]. 나아가 Stang A. [13]은 지금까지 NOS로 관찰연구의 질 수준을 평가한 SR의 도출 결과들은 심각한 오류가 있다고 주장하였다.

한편 일상적으로 섭취하는 식품과 각종 암 발생간의 관련성을 규명하는 식이역학 연구들은 RCT가 사실 불가능하기에, 관찰연구들에 대한 SR을 수행할 수밖에 없다. 이에,

Yang et al. [16]은 기존의 NOS 9가지 항목에, 섭취 에너지 보정여부 항목 (data analysis that used an energy-adjusted residual or nutrient-density model)을 추가하여, 메타분석 대상이 된 논문들의 질적 수준을 평가하고 이를 반영하여 하부군 분석 (subgroup analysis)를 수행하였다. 그런데, Yang et al. [16]이 질적 수준을 평가한 결과를 살펴보면, 코호트 연구 3편 중 3편 모두가 질적 수준이 높은 것 (high quality, HQ)으로 판정한 반면, 환자-대조군 (case-control) 연구 8편 중 2편만이 HQ로 판정하였다. 이렇게 연구설계별로 HQ 판정 차이를 보이는 것은 과학적 설득력이 더 높은 코호트 연구가 더 높은 점수를 받도록 설계된 NOS 속성 때문으로 볼 수 있다 [15].

그렇다면, 메타분석의 대상이 된 관찰연구에 있어 질적 수준의 평가를 위해 NOS 도구를 적용하는 것 대신으로, 연구설계별로 접근하는 것으로 대신할 수 있을까? 즉 NOS 적용을 위해 인력과 시간을 소비하기 보다는 연구설계별로 질적 수준을 통제할 수 있다면 보다 더 합리적이고 효율적일 것이다. 다시 말해서 HQ 분류와 연구설계 간에 일관성을 갖는다면, NOS를 이용한 질적 평가를 대신하여 연구설계별로 하부군분석 (subgroup analysis)를 수행하면 된다. 따라서 본 연구의 목적은 식이역학에 관련한 관찰연구를 대상으로 NOS를 평가도구로 삼은 SR에서, HQ 분류된 것과 코호트 연구간의 분석결과가 동등성 (equivalence)을 갖는지를 알아보는 것이다.

Subjects & Methods

연구대상 논문 검색 및 선정 기준

본 연구의 최종 선정 대상은 일상적으로 섭취하는 식품과 각종 암 발생간의 관련성을 규명한 분석역학연구 논문을 대상으로 한 SR 연구이면서, NOS로 질적 평가를 수행한 뒤 그 결과를 이용하여 하부군 분석 (subgroup analysis)을 수행한 결과를 제시한 것이다. 우선 선정을 위한 논문 목록을 만들기 위하여 PubMed (www.ncbi.nlm.nih.gov/pubmed)를 검색 자료원으로 삼았으며, title, abstract, keyword에서 식품에 해당하는 검색어 – diet, food, fruit, vegetable, meat -와 암발생에 대한 SR이나 메타분석 (meta-analysis)을 의미하는 검색어를 적용하였다. 발표 기간을 2000년 1월부터 2015년 10월까지로 제한하였다.

이렇게 구축한 목록의 논문을 확보한 다음, 내용을 검토하면서 다음의 제외 기준들을 순차적으로 적용하였다. (1) 식이와 암의 관련성을 본 연구가설이 아닌 경우 (2) 관찰연구가 아닌 RCT 연구를 대상으로 수행한 경우 (3) SR을 수행했으나 메타분석은 하지 않은 경우 (4) SR을 수행하면서 질적 평가를 하지 않고 메타분석을 한 경우 (5) SR을 수행하면서 NOS 아닌 다른 도구로 질적 평가한 다음 메타분석을 한 경우 (6) SR을 수행하면서 NOS로 질적 평가를 하였지만 하부군 분석 결과를 제시하지 않은 경우.

관련 정보 추출 및 통계분석

분석 논문들의 질적 평가 수행 여부는 각 논문의 방법에 기술한 내용을 근거로 판단하였으며, 평가한 도구 종류와 HQ 판정 기준을 알아내었다. 체계적 고찰 논문마다 HQ로 평가받은 대상들이 연구설계별로 어떠한가를 알아보기 위해서 코호트 연구와 환자-대조군 연구로 나누어 그 분포를 살펴보고, 선정된 분율(%)간의 통계적 차이를 chi-squared test로 확인하였다.

또한, 하부군 분석으로 HQ군과 코호트 연구에 있어 산출한 summary effect size (sES)와 이의 95% confidence intervals (CI) 정보를 추출하였다. HQ군과 코호트 연구 결과간의 동등성 (Equivalence) 여부는 sES 수치가 null (=1)에 대한 방향성이 유지되고, 95% 신뢰구간에 근거한 통계적 유의성에서 변동이 없는 것을 근거로 판정하였다.

Results

Figure 1은 전자 검색 작업을 통해 최종 분석 대상 논문을 선정한 과정을 나타낸 것이다. 검색식을 적용하여 얻은 목록에서 중복을 제거하였을 때, 371개 논문이 검토대상이 되었다. 이들에서 (1) 연구가설이 식이와 암의 관련성을 본 것이 아닌 경우 256편 (2) RCT 연구를 대상으로 수행한 경우 14편 (3) 체계적 고찰을 수행했으나 메타분석은 하지 않은 경우 6편을 제외하였다. 이후 남은 95편에 있어 연구방법 부분에 NOS를 적용하여 질적 평가를 수행했으며, 그 결과에 따라 하부군 분석 결과가 있는 SR은 14편이었다 [16-29]. 최종적으로 선정된 14편 중 4편 [16-19]은 섭취 에너지 보정여부를 추가한 modified NOS를 사용하였다. Liu et al. [21]를 제외하고는 모두 high quality (HQ) 판정 기준을 7점 이상으로 삼았다. Table 1은 NOS를 적용한 HQ 판정 결정 결과를 코호트 연구와 환자-대조군연구별로 나누어 정리한 것이다. 14편에서 메타분석 대상으로 선정한 89편의 코호트연구와 209편의 환자-대조군연구에서 HQ 로 판정한 경우는 각각 81편 (91%)과 72편 (34%) 으로 통계적으로 유의한 차이를 보였다 ($P<0.01$).

Table 2 는 HQ 군과 코호트 군의 sES와 95% CI를 food item 별로 정리하였다. Wang et al. [22]은 코호트 연구만을 대상으로 수행하였기에 제외하였다. 13편 중 food item 별로 19개의 datasets의 결과를 정리할 수 있었다. 이중 15 datasets 에서 방향성과 통계적 유의성이 동일하였다. 나머지 4 datasets 모두는 방향성에서 변함이 없고 다만 신뢰구간이

넓어지면서 통계적 유의성이 사라진 경우이다.

Table 3은 앞서 Table 2에서 HQ 군과 코호트 군간에 equivalence를 보인 15 datasets에 있어, 코호트군과 환자-대조군의 하부군 분석 결과와 이를 합친 overall sES을 비교하기 위하여 만든 표이다. 코호트 군과 환자-대조군의 sES에서 equivalence를 보인 것은 8 datasets 이며, 이들의 overall sES 역시 equivalence를 보였다. Non-equivalence를 보인 7 datasets에서는 논문 수가 많은 환자-대조군 연구의 결과가 overall sES에 반영되었다.

Discussion

이상의 결과들을 요약하자면, NOS를 사용하여 질적평가를 한 결과에서 대부분의 코호트 연구를 HQ로 판정하였기에 HQ 군과 Cohort 군의 메타분석 결과는 동등성을 보였다. 이를 달리 말하면, 식이역학에 있어 관찰연구를 대상으로 체계적 고찰을 수행할 때, NOS에 의한 질적 평가는 연구방법론에 결정적으로 좌우된다는 것이다. 그렇기에 식이역학의 특성을 고려한 새로운 질적 평가도구가 개발되지 않는 한, 기존의 NOS를 사용한 질적평가보다는 연구설계별로 하부군 분석을 하는 것이 보다 유효하다.

표 2에서 4 datasets 에서 HQ 군과 코호트 군 간에 동등성을 보이지 않았다. 그렇

지만 sES의 방향성은 유지된 채 신뢰구간의 폭에 변동이 생겨서 비동등성으로 평가되었다. 신뢰구간의 폭은 메타분석의 대상논문 수에 따라 변동을 한다는 점에서, 비동등성 결과는 HQ군과 코호트군간의 차이라기 보다는 대상논문 수의 차이에 따른 것으로 해석할 수 있다.

NOS 대신 연구설계별로 얻어낸 결과를 근거로 비동등성을 통제할 수 있는가를 알아보았을 때, 코호트군과 환자-대조군 간의 sES가 동등성을 보일 경우 이를 합친 overall sES 또한 동등성을 보였다. 추가로 Hu et al. [29] 에서 코호트군과 환자-대조군연구간 sES의 신뢰구간이 넓어서 통계적 유의성이 없었지만, 이를 합한 overall sES 신뢰구간은 좁아지면서 통계적 유의성을 확보하였다. 이런 점들을 종합할 때 코호트 군과 환자-대조군의 sES를 각각 구한 다음, 동등성을 보일 경우는 합치는 것이 타당하다는 것을 지지해 준다. 만약 코호트 군과 환자-대조군 간의 sES가 동등성을 보이지 않을 경우는 논문 수에 따라 좌우되므로, 이 경우는 overall sES에 대한 해석에 주의할 필요가 있다.

Colditz et al. [30]은 체계적 고찰에서 있어 이질성 (heterogeneity)을 보이는 이유로 연구설계 (design), 연구수행 수준 (quality of implementation), 노출변수 (exposure), 공변수 (covariates) 로 제시하였다. 식이역학의 메타분석을 수행할 때 코호트 연구과 환자-대조군 연구 모두를 포함하여 선정하기 때문에 연구설계에 의한 이질성이 나타날 수 밖에는 없다 [31,32]. 따라서 동일한 연구설계 내에서 NOS 평가는 의미가 있겠지만, 연구설계를 무시하

고 NOS 평가결과만으로 하부군 분석을 하는 것은 의미가 퇴색되는 것이다.

본 연구를 통해 알게 된 사실은 선정과정에서 NOS가 아닌 자체적으로 개발한 평가기준을 적용한 2편 [33,34] 을 추가하고도 식이역학의 체계적 고찰에서 질적 평가를 수행하고 그 결과를 하부군 분석에 적용한 경우는 16.8% (=16/95 in Fig 1) 라는 점이다. 또한 검색 시작시점을 2000년 1월로 했음에도 불구하고 2006년에 와서야 실제 적용한 논문이 발표되었다는 사실이다 [34]. 이렇게 식이역학의 체계적 고찰에서 질적 평가가 활성화되지 않는 주된 이유는 타당한 측정 도구가 없기 때문이다 [33,34]. 그러나 NOS가 개발된 이후로 이를 활용하게 되었지만, NOS의 HQ 판정은 연구설계에 전적으로 좌우되는 상황임을 본 연구에서 확인하였다. 이는 향후 식이역학에 특화된 질적 평가 도구가 개발되기까지는 연구설계별로 하부군 분석을 하는 것이 합당하다고 본다.

본 연구의 한계점으로는 첫째로, 질적 수준을 평가하는 수준을 알아보기 위하여 PubMed만을 검색 자료원으로 활용했고, 식이항목을 좁혀서 검색식에 적용했다는 점이다. 특히 식이 측정을 통해 이차적으로 알아내는 dietary fat, fiber, vitamin 등에 대하는 제외하였다. 그렇기에, 질적 평가 후 하부군 분석을 한 경우가 16.8% (=16/95 in Fig 1) 라는 수치는 실제보다 과장(over-estimated)되었다고 볼 수 있다. 본 연구결과에 근거해서, NOS 적용을 하지 않더라도 연구 설계별로 하부군 분석을 해서 질적 수준을 통제하는 것에 대한 중요성이 부각되기를 기대한다. 둘째로, NOS를 적용한 연구결과들을 중심으로 코호트군과 환

자-대조군 연구간 sES의 동등성을 비교 검토했다는 점이다. 따라서 연구설계별 하부군 분석 결과를 토대로 식이역학의 메타분석을 위한 질적 수준을 통제할 수 있는가 여부와 그 적용 방법에 대하여는 추가적인 연구가 필요하다.

결론적으로, 식이역학의 메타분석을 위한 질적 평가를 위해 NOS 측정도구를 활용하기 보다는 연구설계별로 하부군 분석을 하는 것이 더 타당하며, 연구설계별로 얻어낸 sES의 동등성에 따라 overall sES를 해석할 필요가 있다는 것이다. 이런 주장은 20년 전에 Greenland S. [35]이 언급한 다음 문장과 일맥상통한다.

"Just as a diet and health study needs to examine the effects of each major dietary factor, quality scoring *should be replaced* by direct regression or stratification on objective quality-related study characteristics, such as *study design (cohort, case-control, etc.),* sources of data (direct interviews, mailed questionnaire, medical records, etc.), and sources of subjects (registry, hospital, etc.)."

ACKNOWLEDGEMENT

I thank Eun Hee Kim in Jeju National University School of Medicine for searching and making the list for review.

REFERENCES

1. Blettner M, Sauerbrei W, Schlehofer B, Scheuchenpflug T, Friedenreich C. Traditional reviews, meta-analyses and pooled analyses in epidemiology. Int J Epidemiol 1999;28:1-9.
2. Shapiro S. Meta-analysis/Shmeta-analysis. Am J Epidemiol 1994;140:771-8.
3. Greenland S. Can meta-analysis be salvaged? Am J Epidemiol 1994;140:783-7.
4. Petitti DB. Of babies and bathwater. Am J Epidemiol 1994;140:779-82.
5. Shapiro S. Is there is or is there ain't no baby? Dr. Shapiro replies to Drs. Petitti and Greenland. Am J Epidemiol 1994;140:788-91.
6. Egger M, Schneider M, Davey Smith G. Spurious precision? Meta-analysis of observational studies. BMJ 1998;316:140-4.
7. Chalmers TC. Problems induced by meta-analyses. Stat Med 1991;10:971-9.
8. Olkin I. Re: "A critical look at some popular meta-analytic methods". Am J Epidemiol 1994;140:297-9.
9. Margetts BM, Thompson RL, Key T, Duffy S, Nelson M, Bingham S, et al. Development of a scoring system to judge the scientific quality of information from case-control and cohort studies of nutrition and disease. Nutr Cancer 1995;24:231-9.
10. Wells GA SB, O'Connell D, Peterson J, Welch V, Losos M, Tugwell P. The Newcastle-Ottawa Scale (NOS) for assessing the quality of nonrandomised studies in meta-analyses. [cited 2016 May 5] Available from: http://www.ohri.ca/programs/clinical_epidemiology/oxford.asp.
11. Deeks JJ, Dinnes J, D'Amico R, Sowden AJ, Sakarovitch C, Song F, et al. Evaluating non-randomised intervention studies. Health Technol Assess 2003;7(27):iii-x, 1-173.
12. Higgins PT, Green S. Cochrane Handbook for Systematic Reviews of Interventions. Wiley-Blackwell: 2008, p.418.
13. Stang A. Critical evaluation of the Newcastle-Ottawa scale for the assessment of the quality of nonrandomized studies in meta-analyses. Eur J Epidemiol 2010;25:603-5.
14. Hartling L, Milne A, Hamm MP, Vandermeer B, Ansari M, Tsertsvadze A, et al. Testing the

Newcastle Ottawa Scale showed low reliability between individual reviewers. J Clin Epidemiol 2013;66:982-93.

15. Lo CK, Mertz D, Loeb M. Newcastle-Ottawa Scale: comparing reviewers' to authors' assessments. BMC Med Res Methodol 2014;14:45.

16. Yang WS, Va P, Wong MY, Zhang HL, Xiang YB. Soy intake is associated with lower lung cancer risk: results from a meta-analysis of epidemiologic studies. Am J Clin Nutr 2011;94:1575-83.

17. Wu QJ, Yang Y, Vogtmann E, Wang J, Han LH, Li HL, et al. Cruciferous vegetables intake and the risk of colorectal cancer: a meta-analysis of observational studies. Ann Oncol 2013;24:1079-87.

18. Wu QJ, Yang Y, Wang J, Han LH, Xiang YB. Cruciferous vegetable consumption and gastric cancer risk: a meta-analysis of epidemiological studies. Cancer Sci 2013;104:1067-73.

19. Zhu H, Yang X, Zhang C, Zhu C, Tao G, Zhao L, et al. Red and processed meat intake is associated with higher gastric cancer risk: a meta-analysis of epidemiological observational studies. PLoS One 2013;8:e70955.

20. Choi Y, Song S, Song Y, Lee JE. Consumption of red and processed meat and esophageal cancer risk: meta-analysis. World J Gastroenterol 2013;19: 1020-9.

21. Liu XO, Huang YB, Gao Y, Chen C, Yan Y, Dai HJ, et al. Association between dietary factors and breast cancer risk among Chinese females: systematic review and meta-analysis. Asian Pac J Cancer Prev 2014;15:1291-8.

22. Wang Q, Chen Y, Wang X, Gong G, Li G, Li C. Consumption of fruit, but not vegetables, may reduce risk of gastric cancer: results from a meta-analysis of cohort studies. Eur J Cancer 2014;50:1498-509.

23. Yang Y, Zhang D, Feng N, Chen G, Liu J, Chen G, et al. Increased intake of vegetables, but not fruit, reduces risk for hepatocellular carcinoma: a meta-analysis. Gastroenterology 2014;147:1031-42.

24. Song P, Lu M, Yin Q, Wu L, Zhang D, Fu B, et al. Red meat consumption and stomach cancer risk: a meta-analysis. J Cancer Res Clin Oncol 2014;140: 979-92.

25. Xin Y, Li XY, Sun SR, Wang LX, Huang T. Vegetable Oil Intake and Breast Cancer Risk: a Meta-analysis. Asian Pac J Cancer Prev 2015;16:5125-35.

26. Wang A, Zhu C, Fu L, Wan X, Yang X, Zhang H, et al. Citrus Fruit Intake Substantially Reduces the Risk of Esophageal Cancer: A Meta-Analysis of Epidemiologic Studies. Medicine (Baltimore) 2015;94:e1390.

27. Wu YC, Zheng D, Sun JJ, Zou ZK, Ma ZL. Meta-analysis of studies on breast cancer risk and diet in Chinese women. Int J Clin Exp Med 2015;8:73-85.

28. Li LY, Luo Y, Lu MD, Xu XW, Lin HD, Zheng ZQ. Cruciferous vegetable consumption and the risk of pancreatic cancer: a meta-analysis. World J Surg Oncol 2015;13:44.

29. Hu J, Hu Y, Hu Y, Zheng S. Intake of cruciferous vegetables is associated with reduced risk of ovarian cancer: a meta-analysis. Asia Pac J Clin Nutr 2015;24:101-9.

30. Colditz GA, Burdick E, Mosteller F. Heterogeneity in meta-analysis of data from epidemiologic studies: a commentary. Am J Epidemiol 1995;142:371-82.

31. Gandini S, Merzenich H, Robertson C, Boyle P. Meta-analysis of studies on breast cancer risk and diet: the role of fruit and vegetable consumption and the intake of associated micronutrients. Eur J Cancer 2000;36:636-46.

32. Xu X, Yu E, Liu L, Zhang W, Wei X, Gao X, et al. Dietary intake of vitamins A, C, and E and the risk of colorectal adenoma: a meta-analysis of observational studies. Eur J Cancer Prev 2013;22:529-39.

33. Bandera EV, Kushi LH, Moore DF, Gifkins DM, McCullough ML. Fruits and vegetables and endometrial cancer risk: a systematic literature review and meta-analysis. Nutr Cancer 2007;58:6-21.

34. Pavia M, Pileggi C, Nobile CG, Angelillo IF. Association between fruit and vegetable consumption and oral cancer: a meta-analysis of observational studies. Am J Clin Nutr

2006;83:1126-34.

35. Greenland S. Invited commentary: a critical look at some popular meta-analytic methods. Am J Epidemiol 1994;140:290-6.
